# Tea Derived Galloylated Polyphenols Cross-Link Purified Gastrointestinal Mucins

**DOI:** 10.1371/journal.pone.0105302

**Published:** 2014-08-27

**Authors:** Pantelis Georgiades, Paul D. A. Pudney, Sarah Rogers, David J. Thornton, Thomas A. Waigh

**Affiliations:** 1 Biological Physics, Department of Physics and Astronomy, University of Manchester, Manchester, United Kingdom; 2 ISIS Facility, Science and Technology Facilities Council, Rutherford Appleton Laboratory, Harwell Oxford, Didcot, Oxfordshire, United Kingdom; 3 Wellcome Trust Centre for Cell Matrix Research, Faculty of Life Sciences, Michael Smith Building, University of Manchester, Manchester, United Kingdom; 4 Strategic Science Group, Unilever Discover, Unilever, Colworth Science Park, Sharnbrook, Bedfordshire, United Kingdom; 5 Photon Science Institute, University of Manchester, Manchester, United Kingdom; Massey University, New Zealand

## Abstract

Polyphenols derived from tea are thought to be important for human health. We show using a combination of particle tracking microrheology and small-angle neutron scattering that polyphenols acts as cross-linkers for purified gastrointestinal mucin, derived from the stomach and the duodenum. Both naturally derived purified polyphenols, and green and black tea extracts are shown to act as cross-linkers. The main active cross-linking component is found to be the galloylated forms of catechins. The viscosity, elasticity and relaxation time of the mucin solutions experience an order of magnitude change in value upon addition of the polyphenol cross-linkers. Similarly small-angle neutron scattering experiments demonstrate a sol-gel transition with the addition of polyphenols, with a large increase in the scattering at low angles, which is attributed to the formation of large scale (>10 nm) heterogeneities during gelation. Cross-linking of mucins by polyphenols is thus expected to have an impact on the physicochemical environment of both the stomach and duodenum; polyphenols are expected to modulate the barrier properties of mucus, nutrient absorption through mucus and the viscoelastic microenvironments of intestinal bacteria.

## Introduction

In recent years there has been growing evidence regarding the beneficial effects on human health of chemicals synthesized in plants called polyphenols, in particular their anti-oxidant properties [1], [2]. Polyphenols are secondary metabolites and are divided into several classes depending on the number of phenol rings they contain and the structural elements that connect these rings, e.g. flavonoids, phenolic acids, stilbenes and lignans [3]. Flavonoids are produced exclusively in plants, in which they play an important role in normal growth development as well as a defence against infections and injuries. Flavonoids are further divided into six subgroups, one being the family of flavanols, to which the focus of this report will be directed, and more specifically to the monomeric form of flavanols known as catechins, the biosynthetic precursor of proanthocyanidins (polymeric flavanols). Catechin (C) and its isomer epicatechin (EC) are the most abundant phenolic molecules found in many types of fruit as well as in red wine and tea [4]. Gallates of epicatechin such as epigallocatechin (EGC) and epigallocatechin gallate (EGCG) are predominantly found in green and black tea, and are differentiated from catechins with respect to the fact that there is at least one gallate ring present in their structure [5].

A number of *in vitro* experiments have revealed the strong radical-scavenging capacity of flavanols, to which their antioxidant properties are mainly attributed. Polyphenols were also demonstrated to alter signalling pathways and subsequent gene expression, thus inducing the secretion of antioxidant enzymes [6]. Through this antioxidant action and the scavenging of reactive oxygen species, such as peroxide and hydroxide radicals, polyphenols help to reduce oxidative damage to biomolecules, which is implicated in the pathology of a number of chronic diseases, such as cancer and cardiovascular diseases (CVD) [2]. Moreover, the use of phenolic compounds as preservatives and supplements in the food industry is common [4].

Even though there have been reports of a negative correlation between the occurrence of certain types of cancer and CVD with the uptake of flavonol-rich foods and beverages, there is little known about the *in* *vivo* efficacy of polyphenols [7], [8]. The interaction of polyphenols with proteins and enzymes during the digestion process is known to limit protein digestibility and thus affect their bioavailability, especially in the case of galloylated catechins [9]. The gastrointestinal (GI) tract is of particular importance, since it is continually exposed to a vast array of reactive species originating from ingested food or generated by chemical reactions of dietary components within the stomach. There, flavonoids could provide protection of the GI tract by scavenging reactive species, but the question is raised whether this depletes the available flavonoid reserve, thus limiting the absorption of them through the small intestine [10].

Most of the absorption of nutrients takes place in the small intestine, where secreted enzymes break down ingested food, so that the necessary nutrients and minerals are absorbed. In order for polyphenols to be absorbed by the body they have to be able to travel through the adherent mucus layer in the small intestine, which acts as a selective barrier that hosts bacteria, while allowing for vital nutritious food molecules to be absorbed. The mucus secretion is a viscoelastic fluid which lines the epithelium of various organs and its function varies between the different mucosal surfaces on which it is found [11]. Mucus is the body’s first line of defence against pathogens, toxins and foreign particles while it also lubricates and reduces friction between mucosal surfaces [12]. More specifically in the GI tract the main role of mucus is protective, both mechanical and chemical. During active digestion, acidic gastric juices are secreted to aid with the breakdown of ingested food, which cause the mucus layer to form a pH gradient through a process of gelation, with the pH ranging from near neutral (pH 7) close to the epithelium to pH 1–2 in the gastric lumen [13], [14]. Mucus thus protects the stomach from auto-digestion.

Mucus is an incredibly complex secretion with its major constituents being water (∼95%), cholesterol, lipids, immunoglobulin, salts, proteins and mucins, the high molecular weight (M_w_ 3–30 MDa) glycoproteins which provide the structural basis of the mucus layer. Two primary forms of mucin exist, the *secreted* and *membrane bound* forms [15]. The secreted mucins are further divided into the *non-polymerising* and *polymerising* forms. This report focuses on polymerising mucins. These large, highly glycosylated proteins are synthesized and secreted as polymers by specialised cells found in the epithelium (goblet cells) and submucosal glands (mucous cells) [11]. Polymerising mucins have at least one large region of the peptide backbone rich in serine, threonine and proline residues, namely the mucin domain, where covalently O-linked oligosaccharides are attached. Polymerising mucins form fibrous assemblies through disulphide bonding at their N- and C- termini, where cysteine-rich domains are found [15]. At physiologically relevant concentrations both the polypeptide backbone and the attached oligosaccharides entangle and reptate in a semi-dilute polymer network, the structural matrix of mucus [16].

Cross-linking in synthetic polymeric molecules has a large impact on their physical properties switching them from a sol (fluid) to a gel (solid) [17]. Intense biophysical research has thus been performed on biochemical cross-linkers, since they can have a profound impact on the micromechanical properties of biopolymers [18]–[21]. The understanding of cross-linkers in glycoprotein or proteoglycan systems is less well developed. There is known to be a switch in the viscoelasticity of both aggrecan and stomach mucin as a function of pH due to the modulation of the number of active cross-linkers [22], [23]. Here, we use isolated porcine gastric (Muc5ac) and duodenal (Muc2) mucins as models for human mucins to provide evidence that polyphenols from tea act as cross-linkers of both stomach and intestinal mucins. As well as modulating the availability of the polyphenols for absorption, cross-linking will profoundly modify the viscoelasticity of the boundary layers attached to both the stomach and the duodenum, which are inhabited by swimming bacterial microflora. [24]. We thus believe the activity of polyphenols that act as cross-linkers could have important implications for human health.

## Materials and Methods

### 2.1 Mucin purification

Gastric and duodenal mucins were used as a model system for gastrointestinal mucus, isolated from gastrointestinal mucus as previously reported. [16] Animal tissue was obtained post-mortem from Nixon’s farmshop abattoir. No pigs were euthanized specifically for this study. Crude mucus was scraped from the epithelium of stomachs and duodena, homogenised in 0.2 M NaCl buffer with proteinase inhibitors and 0.2% w/w sodium azide. Two-step CsCl density gradient centrifugations (starting densities 1.4 g/ml and 1.5 g/ml were chosen for the first and second step respectively) followed the previous step and the mucin rich fractions (identified by Schiff reagent/PAS staining and UV absorption) were pooled, extensively dialysed against dd H_2_O and lyophilised [14], [25]. The mucin samples were found to be of the expected molecular weight (2–200 MDa) using size exclusion chromatography with multi-angle light scattering (SEC-MALS) [26].

### 2.2 Particle Tracking Microrheology (PTM)

Lyophilised mucins were solubilised in a 10 mM phosphate buffer at pH 7 and after equilibrium was reached (48 hrs) they were treated with lyophilised polyphenols or tea extracts. The polyphenols used in these studies were EC, EGCG, black and green tea extracts and EGCG-rich green tea extract (SI text).

Carboxyl-activated polystyrene microspheres (505 nm, Polysciences Inc.) were embedded in the mucin/polyphenol solutions. These acted as probe particles and their thermal motion was recorded using an inverted Olympus IX-71 microscope equipped with a 100× immersion oil objective (f/2.0) and a FastCam 1024-PCI fast camera (10^5^ fps). The setup was placed on an active optical table to minimise external vibrations. The probe particles’ trajectories were extracted from the videos using a parallel version of Polyparticletracker [27], [28]. The diffusion of the beads was previously shown to be unchanged in mucin solutions over a large range of frequencies after coating with polyethylene glycol (PEG), which provide a barrier against mucin adsorption [16]. Furthermore, the addition of EGCG in a solution containing just probe spheres was found not to alter the diffusion coefficient of the probe spheres.

The trajectories of the probe spheres were used to calculate their mean square displacement (MSD) as a function of lag time (τ). For a sphere suspended in a medium, the exponent (α) of a power law fit to the MSD as a function of lag time is an indication of the nature of the medium, ie. from, 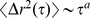, α = 1 corresponds to a purely viscous liquid, whereas α = 0 corresponds to a purely elastic material. In the case where α lies in the range 0<α<1, the material’s response is dependent upon the time scale of the measurement relative to a characteristic time of the material, namely the relaxation time, τ_rel_. [29].

The time dependent compliance (*J(τ)*) is a standard viscoelastic measurement (units of Pa^−1^), which can be computed from the MSD (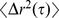) of each bead with radius a using [30].
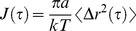
(1)where kT is the thermal energy. By using the Maxwell model for linear viscoelasticity, the viscosity (*η*) and elastic shear modulus (*G*) of the sample can be extracted using a fit of



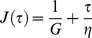
(3)Additionally, the viscosity and the elastic shear modulus are related to the relaxation time by 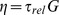. A previous study by our group indicated good agreement between particle tracking microrheology results for the linear viscoelasticity of gelling mucins and literature values for the bulk rheology made using conventional rheometers. [16] The PTM experiments were repeated three times and the ensemble averaged MSDs presented in the relevant section were obtained from averaging over more than 1000 probe spheres per sample, which were imaged over randomly chosen regions within the bulk of the solution i.e. at least 10 µm away from the walls of the sample cell.

### 2.3 Small Angle Neutron Scattering (SANS)

For SANS experiments, D_2_O was used instead of H_2_O, to provide optimum contrast between the mucins and the buffer. Due to limited sample and beam time availability the experiments were performed once for each sample (each sample requires one hours beam time), but they were in agreement with previous SANS experiments with mucin [31] and accurately fulfilled internal consistency checks on the scattered intensity with mucin concentration. Due to their minimal requirements on sample preparation, SANS experiments are typically high reproducible. SANS measurements were performed at the SANS2D diffractometer in ISIS, Rutherford Appleton Laboratory, Didcot, UK on the newly commissioned second high flux target station. [32] This allowed us to probe a q range of 0.005–0.59 Å^−1^, using the 1 m^2^ detector at a distance of 4 m from the sample. q is defined as 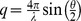, where θ is the scattering angle and λ the incident neutrons’ wavelength [33]. Porod plots (log(I(q)) vs log(q)) allowed us to extract the fractal dimension (n) of the scattering objects (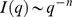). For phase separated semi-dilute gels, the scattering intensity from the polymer gel is expected to be larger than that of the corresponding solution. In order for the overall scattering profile to be described adequately, I(q) is broken down into two components:
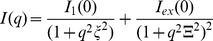
(1)where the first term is the Ornstein-Zernicke equation in which ξ is the *semi-dilute correlation length* and I_1_(0) is the zero-q extrapolated intensity of the polymer solution. The second term is the Debye-Bueche function, which describes the excess scattering originating from the higher-order structured polymer gel. Ξ is the average thickness of the micro phase separated aggregate and 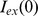 is the amplitude of the heterogeneity [34]. Such a model has previously been shown to provide a good description of SANS data from pH switched mucin gels [26].

## Results

### 3.1 Mixtures of purified gastric mucin solutions with polyphenols

**Figure 1:**
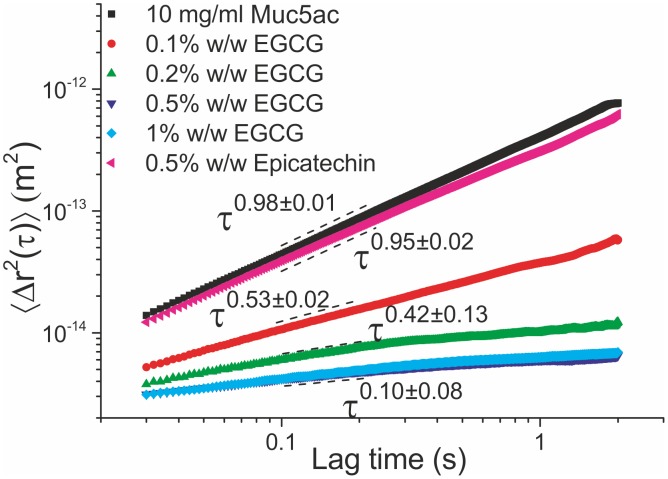
The ensemble averaged MSD curves as a function of lag time obtained from a 10 mg/ml Muc5ac solution before and after treatment with EGCG and EC. Treatment with 0.5% w/w EC had little effect on the rheological properties. Treatment with as little as 0.1% w/w EGCG induced a dramatic effect, causing the mucin solution to become viscoelastic. At concentrations above 0.5% w/w EGCG the solution has undergone a sol-gel transition and no further change could be detected using PTM.

The effect of the addition of polyphenols on the viscoelastic characteristics of purified mucin solutions was investigated using particle tracking microrheology. Mucins used in this study were previously demonstrated to have the gelling properties expected of native mucins, that commercially available mucins lack [16]. Fig. 1 shows the ensemble averaged MSDs of the probe spheres as a function of lag time obtained from a 10 mg/ml Muc5ac solution before and after treatment with 0.5% w/w EC, EGCG and tea extracts. The maximum polyphenol concentration used for each compound reflects its solubility limit in water.

The viscosity, elastic shear modulus and the resulting relaxation times of the aforementioned solutions are shown in Table 1 (SI text). Treatment with 0.5% w/w EC resulted in a very small change in viscoelasticity of the initial stock solution; the viscosity was slightly increased to 14.4±0.6 mPas from 12.0±0.3 mPas, whereas the fitted power exponent was found to be 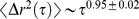, from 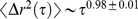.

Contrary to the minimal effect of EC, treatment with 0.1% w/w EGCG triggered a transition to a viscoelastic fluid, evident by the 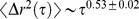 fitted power law, and considerable increase in viscosity and elastic shear modulus. Additionally, two populations of embedded microspheres were observed (Fig. S1 in File S1), one trapped in the gel network and one diffusing in regions of lower viscosity. Upon treatment with 0.2% w/w EGCG there was a 10-fold increase in viscosity observed compared with the untreated solution and the relaxation time was found to be 427±51 ms, which is of the order expected from weak physical hydrogels. In conjunction with the plateau of the MSD curve at longer lag times, it suggests that the solution underwent a sol-gel transition. Further increases in EGCG concentration induced further increases in the viscoelasticity of the solutions.

**Figure 2:**
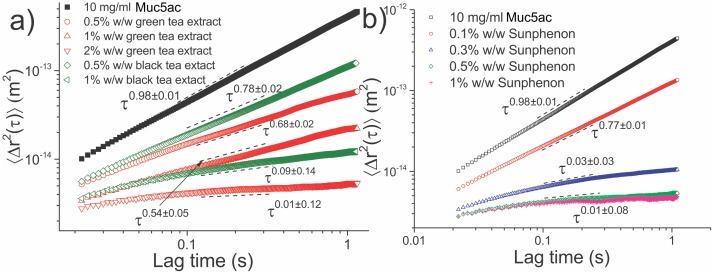
The ensemble averaged MSD curves obtained from a 10 mg/ml Muc5ac solution before and after the addition of (a) green and black tea extracts and (b) the EGCG-rich green tea extract Sunphenon 80SK. The addition of up to 1% w/w green tea extract caused the solution to become viscoelastic, whereas at 2% w/w the solution has undergone a sol-gel transition. Similarly, at 0.5% w/w black tea extract the solution is viscoelastic, whereas at 1% w/w the solution appears to have transformed into a weak gel. The addition of 0.1% w/w Sunphenon extract caused the purely viscous liquid to become viscoelastic, with a power law of 0.77±0.01. At a concentration of 0.3% w/w Sunphenon extract the solution undergoes a sol-gel transition, evident by the 0.03±0.03 power law exponent of the MSD curve. At 0.5% w/w Sunphenon there is an increase in the viscoelastic characteristics of the gel and no further change could be detected using PTM at higher concentrations.

Additionally, the Muc5ac solutions were treated with black and green tea extracts, shown in Fig. 2a. At 0.5% w/w green tea extract, the MSD’s power exponent was found to be 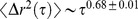, suggesting another transition to a viscoelastic solution, with a 10-fold increase in viscosity and elastic shear modulus. Further increase in the concentration of green tea extract resulted in the formation of a stronger polymer network, when finally at 2% w/w the solution undergoes a sol-gel transition, demonstrated by the 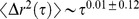 fitted power law. Similar heterogeneities were observed as with the EGCG treated solutions (Fig. S2 in File S1) i.e. two populations of embedded sphere motility were found. The relaxation time of the gel was found to be 1.6±0.1 s, which is of the order expected for associating physical hydrogels.

At a concentration of 0.5% w/w black tea extract there is again a transition from a purely viscous liquid to a viscoelastic material, with a power law exponent of 0.75±0.01. Treatment with 1% w/w black tea extract induced a sol-gel transition and resulted in a weak gel, as implied by the fitted power law of 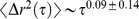. The viscosity of the solution was measured to be 444±7 mPas, its elastic shear modulus is 0.85±0.02 Pa and its relaxation time is 522±15 ms.

In addition to the aforementioned tea extracts, an EGCG-rich green tea extract (Sunphenon 80SK) was also used to investigate its effects on the rheological characteristics of Muc5ac solutions, shown in Fig. 2b. At a concentration of 0.1% w/w, the solution becomes viscoelastic, with η = 36.5±0.2 mPas, G = 0.69±0.02 Pa and τ_rel_ = 53±2 ms. Further addition of Sunphenon has caused the solution to gel, evident from the 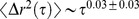 power law and the plateau of the MSD curve at long lag times. Furthermore, the viscosity was measured to be a 10-fold increase over that of the solution containing 0.1% w/w of Sunphenon compound. Its elastic shear modulus was found to be 0.92±0.01 Pa and its relaxation time is 537±11 ms. At a concentration of 0.5% w/w there is a further increase in the viscoelastic properties of the gel, namely to η = 2.02±0.1 Pa, G = 1.27±0.01 Pa and τ_rel_ = 1.59±0.08 s. At this point the gelled solution was at the limits of the sensitivity of the PTM technique, as demonstrated by the MSD curve obtained from a 1% w/w treated gel, which coincides with that of the lower concentration of the compound. The probe spheres’ motion was barely detectable using our setup, and produced a noisy MSD curve. Fitting the resulting compliance curve we measured the viscoelastic parameters to be η = 3.5±0.3 Pas, G = 1.3±1 Pa and τ_rel_ = 2.6±0.3 s.

### 3.2 Purified soluble Muc2 mucin and polyphenol particle tracking microrheology

**Figure 3:**
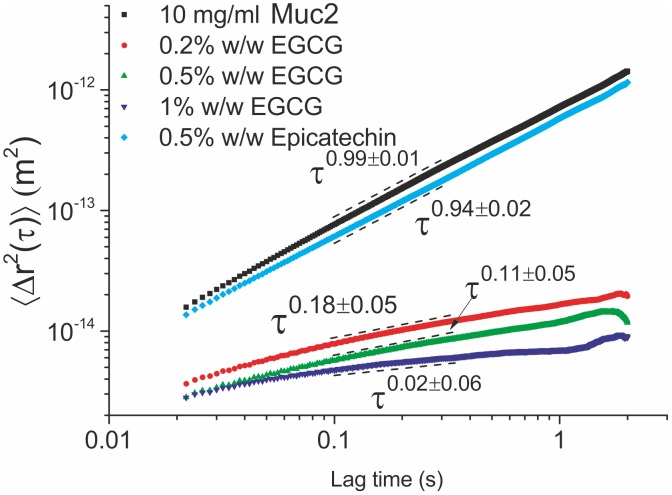
The ensemble averaged MSD curves as a function of lag time for Muc2 solutions treated with (a) EC and EGCG and (b) green and black tea extracts. There is little change in the viscoelasticity of the mucin solution when treated with EC. Treatment with 0.2% w/w of EGCG induced a sol-gel transition, whereas treatment with 0.5% w/w or higher EGCG results in a stronger gel.

The ensemble averaged MSD curves from probe particles in 10 mg/ml Muc2 solutions treated with EC and EGCG are shown in Fig. 3 and the measured viscoelastic properties of the solutions are presented in Table 2 (SI text).

The stock 10 mg/ml Muc2 solution was found to be purely viscous, as confirmed by the fitted power law exponent of 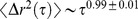. Treatment with 0.5% w/w EC induced a small change in the observed viscoelasticity with a power law fit of 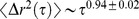, and a small increase in viscosity from 6.7±0.3 mPas to 8.9±0.4 mPas.

Treatment with 0.2% w/w EGCG induced a sol-gel transition in the mucin solution with an associated increase in viscosity and elastic shear modulus, evident by the 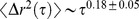 power law. As with Muc5ac, there were bimodal regions of lower and higher viscosity identified within the sample (Fig. S3 in File S1). Further increase in the extract’s concentration to 0.5% and 1% w/w resulted in a stronger gel, the fitted power laws were 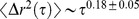
and 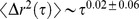 respectively. Additionally, the distribution of power law exponents is clearly skewed towards zero.

**Figure 4:**
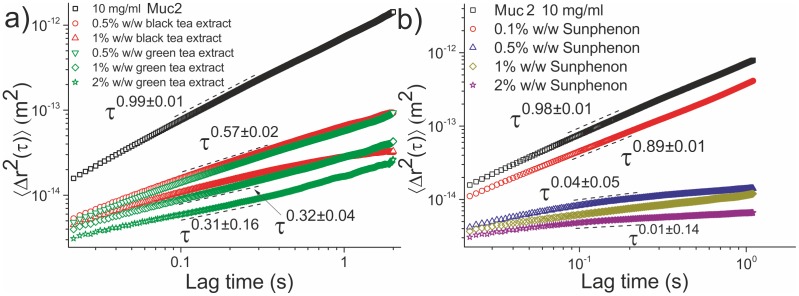
The ensemble averaged MSD curves as a function of lag time for a 10 mg/ml Muc2 solution treated with (a) black and green tea extracts and (b) the EGCG-rich green tea extract Sunphenon 80SK. The addition of green tea extract causes the solution to become viscoelastic, with a monotonic correlation observed between the extract’s concentration and viscoelasticity. Similarly, 0.5% and 1% w/w black tea extract cause the solution to become viscoelastic. At a concentration of 0.1% w/w Sunphenon extract the solution becomes viscoelastic, whereas at a concentration of 0.5% w/w the solution undergoes a sol-gel transition. Increasing the concentration of the extract results in a stronger gel, which is at the limits of what PTM can detect.

An increase in the concentration of the two tea extracts leads to stronger viscoelastic solutions, demonstrated by the decreasing absolute MSD values of the embedded probe spheres and a decrease of the power law exponent of the curves show in Fig. 4a. Gradual increase in the concentration of green tea extract to 2% w/w leads to the formation of a stronger viscoelastic network, as depicted by the monotonic correlation between the concentration of the extract and the measured properties of the solutions.

Treatment with black tea extract had similar results as with the green tea extracts. The power laws at 0.5% and 1% w/w were found to be 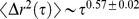 and 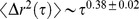 respectively, that indicate the formation of a stronger viscoelastic network with increasing black tea concentration. A direct correlation between the concentration of the extract and the measured viscoelastic properties of the treated solutions is again observed, as shown in Table 2 (SI text).

As with Muc5ac, the EGCG-rich Sunphenon extract was used in PTM experiments (Fig. 4b). At a concentration of 0.1% w/w of the compound, the solution is viscoelastic, as shown by the 0.89±0.01 power law exponent; individual beads’ MSDs are presented in Fig. S4 in File S1. At a concentration of 0.1% w/w Sunphenon, there were two phases observed in the solution, a gelled and an ungelled phase. Its viscoelastic properties were measured to be η = 13.1±0.1 mPas, G = 0.71±0.03 Pa and τ_rel_ = 18±1 ms, values typical for a weak viscoelastic fluid. However at 0.5% w/w the solution undergoes a sol-gel transition. The power law fit was found to be 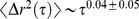, also observed in the individual beads’ power exponents histogram (Fig. S4 in File S1). The viscosity of the solution was found to be greatly increased compared to the lower concentration of Sunphenon extract treated solutions, and its relaxation time was of the order of hundreds of ms, as is the case with weak hydrogels. Further increases in the compound’s concentration resulted in stronger gels, shown by the decrease in the absolute values of the MSD curves and increased viscosity. Finally, at 2% w/w the viscoelastic parameters were found to be η = 1.30±0.08 Pas, G = 1.12±0.08 Pa and τ_rel_ = 1.16±0.12 s.

As with Muc5ac, the stock 10 mg/ml Muc2 solution was also treated with the EGCG-rich green tea extract Sunphenon 80SK, producing comparable results (Fig. S3 and S4 in File S1).

### 3.3 Small Angle Neutron Scattering results

**Figure 5:**
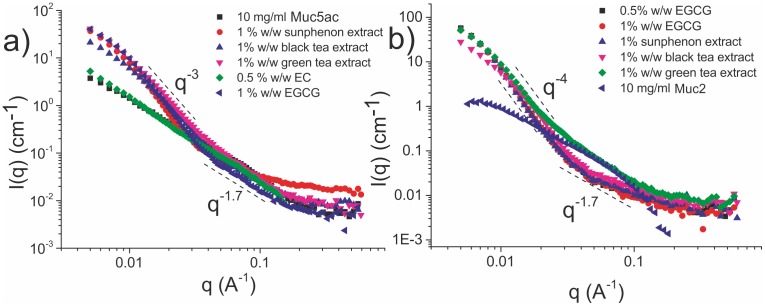
The scattering profiles of 10 mg/ml (a) Muc5ac and (b) Muc2 solutions treated with EC, EGCG and tea extracts. In both types of mucin, the scattering profile of the stock solution is described in its entirety with a I(q)∼q^−1.7^ power law and the addition of 0.5% w/w EC had very little impact on it. Treatment with 0.5%, 1% w/w EGCG and the tea extracts induced a large increase in the low-q regime in both types of mucin, a sign of formation of large length scale structures (>10 nm) within the sample. In the intermediate q-range there is a universal I(q)∼q^−1.7^ power law, observed in all the solutions. In the low-q range, there was a I(q)∼q^−3^ power law observed for treated Muc5ac solutions, whereas a I(q)∼q^−4^ power law was observed in Muc2 solutions.

In addition to our microrheological studies of mucin/polyphenol solutions, the effects of polyphenols on the structure of mucin networks were examined using SANS. Fig. 5 shows the normalised scattering profile as a function of momentum transfer (q) of a 10 mg/ml Muc5ac solution treated with 0.5% w/w EC, 1% w/w EGCG and the tea extracts, compared to that from the initial stock mucin solution. The fractal dimension for the untreated Muc5ac mucin solution extracted from the Porod plot of the scattering profile was 1.67±0.01 over the whole q-range examined, which is a result expected for linear swollen polymers; that is polymers in good solvent conditions [35]. Treatment with EC did not induce a considerable change in the scattering profile of the mucin solution. However, addition of 1% w/w EGCG had a dramatic effect on the intensity of the scattering profile in the low-q range. The absolute intensity was increased by a factor of more than 10 and a fractal dimension of 3.54±0.02 was observed in the Porod plot at low q, whereas a fractal dimension of 1.64±0.03 was observed in the intermediate-q range; which lies between the two extremes at q^−2^ and q^−1^, (i.e. a coil and a rod respectively).

Furthermore, 1% w/w EGCG-rich green tea extract (Sunphenon) induced a 20-fold increase in scattering intensity in the low-q regime for the 10 mg/mL Muc5ac solution, whilst the fractal dimensions observed were 3.14±0.07 and 1.71±0.07 in the low- and intermediate-q regime respectively. Black tea extract had similar effects on the intensity in the low-q regime, albeit slightly lower compared to green tea extracts. The fractal dimensions observed in the Porod plot of the scattering profile were 2.94±0.02 and 1.61±0.03 in the low- and intermediate-q regimes respectively. Green tea extract exhibited a similar increase in absolute intensity in the low-q range, with fractal dimensions 3.31±0.04 and 1.85±0.03 in the low- and intermediate-q range respectively.

The scattering profiles of a 10 mg/ml Muc2 solution treated with purified 0.5% and 1% w/w EGCG compared to the untreated solution are shown in Figure 3b. A fractal dimension of 1.65±0.02 was observed in the entirety of the accessible q-range for the untreated Muc2 solutions. Treatment with 0.5% and 1% w/w EGCG produced similar scattering profiles as with Muc5ac, with more than a 10-fold increase in absolute scattering intensity in the low-q regime. The fractal dimensions observed for the 0.5% w/w EGCG were 3.98±0.08 and 1.73±0.09 in the low- and intermediate-q regimes respectively, whereas for the 1% w/w treated solution were found to be n = 3.95±0.07 and n = 1.70±0.09 respectively.

Addition of all of the aforementioned EGCG containing tea extracts induced a dramatic increase in the scattering intensity in the low-q regime. The fractal dimensions observed for the EGCG-rich green tea extract (sunphenon) were 3.97±0.06 and 1.71±0.01 in the low- and intermediate-q regimes respectively. The same parameters for the 1% w/w black tea treated solution were 3.94±0.07 and 1.75±0.09 in the low- and intermediate-q regimes respectively, whereas for the green tea extract they were found to be 3.78±0.04 and 1.76±0.06 respectively.

By fitting the Ornstein-Zernicke function (the first term on the right hand side of eq. (1)), the semi-dilute correlation lengths for the untreated mucin solutions were found to be 14.0±0.9 Å and 9.3±1.3 Å for Muc5ac and Muc2 respectively. Polyphenol treated mucin solutions were fitted with Eq. (1) from which Ξ, the average thickness of the phase separated aggregates, was calculated (Fig. S7 in File S1). Table 3 (SI text) shows the parameters calculated from the SANS scattering profiles.

## Discussion

The effects on the viscoelasticity and structure of tea derived polyphenols on gastrointestinal mucin solutions were investigated using PTM and SANS. There is very little available information in the literature regarding the molecular interactions between the two types of molecule. Most of the available reports have investigated the interactions of whole saliva or proline rich proteins (PRPs) with polyphenols, which is believed to be the basis of the astringent feeling experienced after the consumption of certain fruits and tea [36]–[39]. Here, we used biochemically well-characterised pig gastric (Muc5ac) and duodenal (Muc2) mucins as model equivalents for human mucins to study their interaction with purified catechins and tea extracts. The mucin samples used in this study were prepared from crude mucus scrapings and were carefully isolated under low temperatures and in the presence of proteinase inhibitors and sodium azide without the use of reducing or chaotropic agents to preserve the native conformation of the molecules. This method of mucin extraction unavoidably excludes the extraction of a fraction of mucins present in the “insoluble” thicker inner layer of mucus. Extraction of this layer is not possible without the use of chaotropic agents. However our samples exhibit the expected molecular weight distribution expected from mucins (2–200 MDa) and they were previously demonstrated to have the associative characteristics of native mucins [16], [26]. This large range of molecular weights is expected for mucins; secreted oligomers are made up from variable numbers of monomers which can be further segregated due to extracellular degradation and sample preparation [16], [40], [41]. The size of the mucin oligomers can potentially affect the viscoelasticity scaling of the solutions, with longer molecules’ viscoelasticity scaling faster. We expect this variability in size to have a small contribution in this case, due to the large number of cross-links created and their dominant effects on the viscoelasticity and the neutron scattering.

Treatment of a 10 mg/ml Muc5ac solution with as little as 0.1% w/w of EGCG caused a dramatic effect on its rheological characteristics, inducing a transition from a purely viscous liquid to a viscoelastic material with a considerable increase in both viscosity and elastic shear modulus. At any higher concentration of EGCG, the solution undergoes a sol-gel transition, since at 0.2% w/w the MSD of the probe spheres reach a plateau and the long relaxation time (427 ms) indicates a weak gel has been formed. Further increases in EGCG concentration causes the solution to become a stronger gel, since the viscosity, elastic modulus and relaxation time of the material were all found to increase. Above 0.5% w/w ECGC the solution is at the limits of PTM technique’s sensitivity and little change can be detected, since the thermal collisions on the probe spheres do not induce measurable displacements. In contrast, addition of epicatechin to the MUC5AC solution induced very little, if any, change to the rheological properties of the solution, with the ensemble averaged MSDs from probe spheres in the two solutions almost coinciding. Treatment of 10 mg/ml MUC2 solutions with the same compounds resulted in very similar results, with the solution gelling above a concentration of 0.2% w/w EGCG, whereas very little change resulted from treatment with EC.

The aforementioned findings are in agreement with what was expected from the literature [42]. EGCG has been shown to adsorb and form multilayers with commercially available (non-gelling) Orthana mucin, but no aggregation was reported. Furthermore, an increase in the size of the hydrophobic globules at the two ends of the Orthana molecules was reported, when EGCG was added, with a two-fold increase in the globules’ radius [42]. Also, EGCG has been demonstrated to strongly interact with β-casein and proline rich proteins (PRPs) in human whole saliva, forming precipitates of aggregated protein complexes [36], [43].

**Figure 6:**
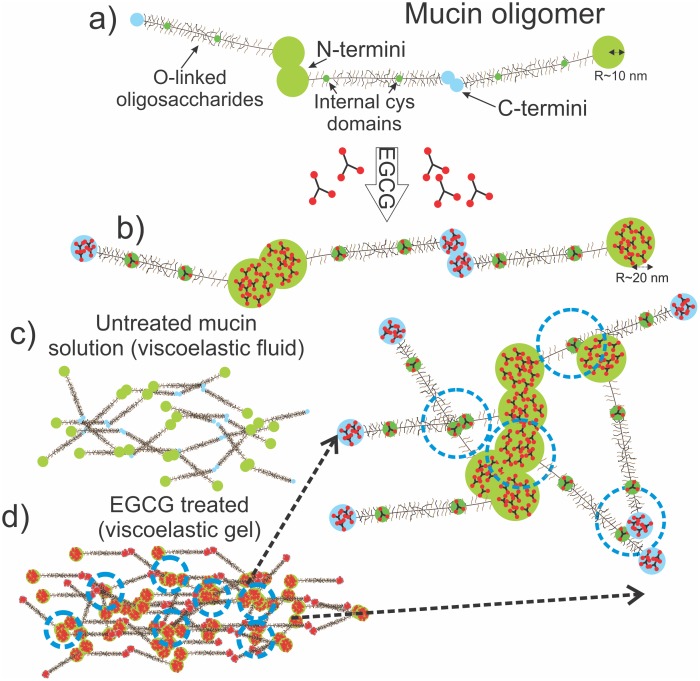
A schematic diagram of the proposed mechanism that leads to gelation of mucin solutions upon treatment with EGCG. (a) Mucins are naturally secreted as oligomers, which are connected end-to-end through disulphide bonding at the two termini. The amount of internal cys- domains along the peptide backbone differs depending on the mucin type. (b) Clusters of EGCG bind to the exposed domains by hydrogen bonding on amino acids such as valine or leucine, which increases their size and creates sites for cross-links with other mucin molecules. (c) Untreated mucin solutions at pH 7 form semi-dilute unentangled networks, which behave as a purely viscous liquid. (d) Mucin/EGCG complexes in solution form cross links (blue circles), which form aggregates and given enough EGCG concentration eventually form a gel. (e) A magnification of the enlarged domains which act as crosslinks between mucin oligomers. The exposed domains along the peptide backbone can potentially contribute to the cross-linking process.

The non-glycosylated regions of both Muc5ac and Muc2 do not contain proline-rich sequences, which therefore does not explain the dramatic effects of EGCG on the structure and microrheology of mucin solutions. However other exposed amino acids located on the two termini at each end of the molecule and the cys domains along the peptide backbone could also act as sites for EGCG binding through hydrogen bonding. Additional EGCG self-associations could allow further EGCG adsorption onto mucin oligomers, leading to multilayer adsorption and an increase in the size of the interacting domains (Figure 6b) [42]. As the concentration of EGCG increases, complexes of the molecule formed at the two termini and the cys domains, which cross-link with other mucin molecules and create aggregates. The formation of large scale aggregates in the solution was observed in SANS experiments, which was characterised in terms of a two phase separated system. Further aggregation eventually leads to a sol-gel transition which has a profound effect on the viscoelasticity of the mucin solution. The complexation and the increase of the hydrophobicity of the molecule (due to masking of the hydrophobic globular ends of the molecule) were apparent after addition of EGCG, as shown in optical microscopy images that show gelled samples in Figure S5 in File S1.

The formation of large structures, i.e. aggregates, within the solution when EGCG is added is also apparent in the scattering profiles of the treated solutions. A single fractal dimension (n) is observed throughout the accessible q-range for the untreated Muc5ac and Muc2 solutions, which is very close to 1.69, expected for swollen linear polymers (by mass this would be expected to reflect the conformation of the carbohydrate side-chains) [44]. Solutions treated with EGCG exhibit a large increase in the intensity of the scattering curve at low-q which, implies the formation of large structures within the solution, as one would expect from a gel. Moreover, two distinct power laws could be observed, with the transition occurring at q∼0.03 Å, which corresponds to a length scale of ∼50 nm. This is in line with the complexation of enlarged globules (r∼20 nm) to form aggregates and eventually a percolated gel network [42].

The untreated mucin solutions’ SANS intensity profiles can be described by a simple Ornstein-Zernicke function, while the upturn of the intensity scattered by polyphenol treated solutions’ at low-q requires the addition of the Debye-Bueche term, which describes a two-phase separated system with sharply demarcated concentration boundaries. The small-scale correlation length (ξ) extracted by fitting the Ornstein-Zernicke function to the untreated solutions’ profile was 14.0±0.9 Å and 9.3±1.3 Å for Muc5ac and Muc2 respectively. This length scale corresponds to scattering from the carbohydrate side-chains, which are attached to the peptide backbone and are expected to have very little or no interaction with phenolic molecules [26]. The same parameter for polyphenol treated mucin solutions was found to be unchanged for both types of mucins when compared with the untreated samples (slight variations are expected due to the highly complex nature of these biomolecules).

**Figure 7:**
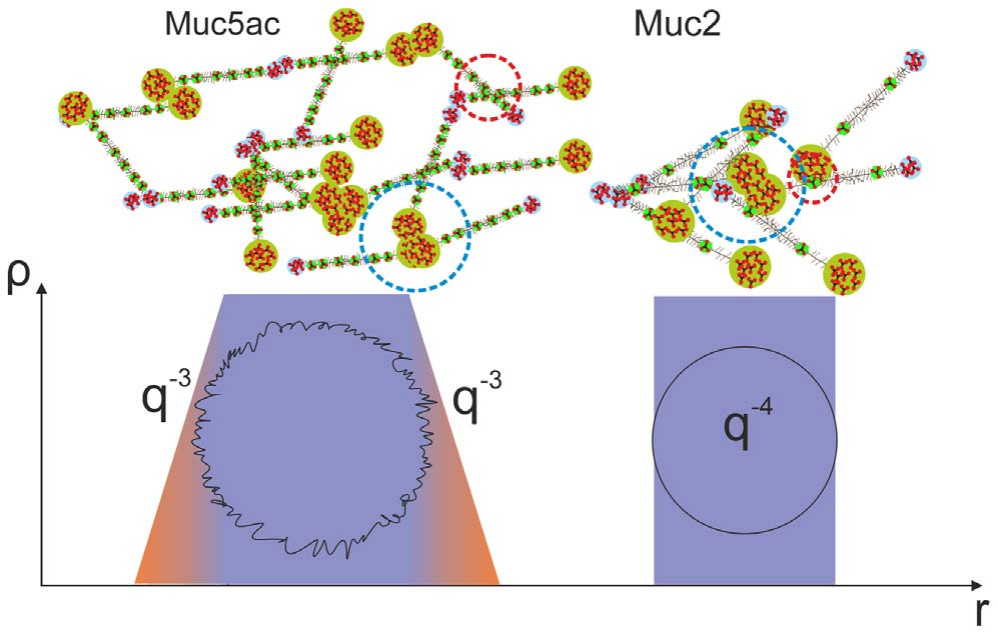
A schematic representation of the cross-linking process after the addition of EGCG to Muc2 and Muc5ac solutions. The Muc2 molecule has only two cys-domains along its peptide backbone, which are saturated by EGCG easier than Muc5ac, which has at least 8 cys-domains. The presence of a large number of cys domains provides for a larger number of available sites for cross-linking, which lead to the formation of smaller aggregates compared to Muc2 (observed with SANS). Additionally, the presence of less cys-domains in the molecular structure of Muc2 leads to a more complete phase separation within the solution, with distinct boundaries (q^−4^ scattering), whereas Muc5ac has less distinctive boundaries between phases (q^−3^ scattering) as a result of a number of cys-domains remaining uncomplexed. Blue circles represent aggregation at the two termini of the mucin molecules, whereas red circles show cross-linking occurring at the cys-domains of the molecule.

It was previously shown that for two-phase systems ξ is expected to get smaller with concentration and Ξ to get larger, since Ξ probes the average thickness of the micro phase separated aggregate. It is also expected that Ξ>>ξ. [34] Indeed, a monotonic increase is observed between Ξ and the relaxation time of the solutions. As shown in Table SI3, the pure EGCG and EGCG-rich green tea extract treated Muc5ac solutions were observed to have larger average spacing between inhomogeneities when compared to the black tea extract treated ones. Analogous observations are made from the Muc2 treated solutions. EGCG and Sunphenon extract treated Muc2 solutions were found to have considerably larger Ξ when compared to the black tea extract treated solutions i.e. they have large aggregate structures. Similar conclusions result from the comparison of the excess intensity at low-q (I_ex_(0)) between the treated solutions. This observation is in line with the relative galloylated content of the mixtures used. Black tea has a lower concentration of EGCG and other galloylated catechins compared to green tea extracts, thus it is expected to have milder effects on the structure and rheology of mucin solutions. Muc2 solutions were observed to have undergone a more complete phase transition compared to Muc5uc solution (q^−4^ dependence at low-q compared to q^−3^ for Muc5ac, Fig. 7). This is also shown by the considerably larger extrapolated zero-q excess scattering (I_ex_(0)) derived using Eq. (1) from Muc2 solutions compared with Muc5ac.

Figure 7 shows a schematic representation of the difference in the structure of the two mucin molecules examined and how the number of cys-domains along the peptide backbone might influence the overall gelation process after the addition of galloylated catechins. The two types of mucins share common C- and N- terminal structures, but differ in the number of cys- domains interspersed throughout the peptide backbone, to which the different power laws observed in the low-q regimes of the SANS profiles could potentially be attributed (q^−3^ dependence in the low-q range of Muc5ac treated with EGCG and q^−4^ for Muc2).

In addition to the two compounds containing only a single polyphenol molecule (EGCG and epicatechin), we also examined the effects of black and green tea extracts, which are also good sources for a variety of flavanols. Both types of mucin undergo a sol-gel transition when 1% w/w green tea extract and 0.5% w/w of EGCG-rich green tea extract are added. Muc5ac produced slightly stronger gels at the same concentration compared to Muc2. A possible explanation for this observation would be the presence of cys-domains knots along the peptide backbone on Muc5ac molecules (at least 8) compared to just two along the backbone of Muc2. This could potentially provide an increased number of available sites for EGCG bonding and aggregation (Fig. 7 and Fig. S6 in File S1). Furthermore, upon addition of black tea extract both types of mucin formed gels at a concentration of 1% w/w, and again Muc5ac formed a stronger gel compared to Muc2.

Polyphenols are abundant in plant derived food and beverages; chocolate and black tea are especially good sources since their total phenolic content can approach 1 g per serving [45]. Furthermore, the galloylated catechin content of green tea varies depending on brand and origin and its estimated to be in the 35–110 mg range [46] and it is estimated that the average stomach is lined with around 100 ml of mucus [40]. Given these estimates, the consumption of 4–5 cups of tea per day, typical in certain countries and diets, could provide sufficient amounts of galloylated catechins to induce a change in the rheology of the adherent mucus layer in the stomach and the small intestines i.e. induce a sol-gel transition of the mucins. Indeed, a negative correlation was found in the occurrence of certain types of cancer and cardiovascular disease and the consumption of an average of 5 cups of tea per day in previous trials [8]. However though the crosslinking of mucins by galloylated catechins induces significant effects in the viscoelasticity of ex-vivo purified mucin solutions, the same might not be true for in-vivo mucus secretions. Mucus is a complex secretion, with several constituent proteins which can potentially interact with galloylated catechins, thus the effect could potentially not be as marked in-vivo as demonstrated with ex-vivo purified mucins. Additional research in vivo is clearly required to address this issue.

The observed interactions of EGCG with mucins and the lack of interaction of EC with the mucus layer is in line with clinical investigations of the degree of absorption of these molecules by the human body. Investigations of ileal fluid and urine from healthy subjects who ingested a polyphenol cocktail rich in both EC and EGCG revealed that EC is absorbed through the small intestine, whereas no 3-0-galloylated flavanols or their metabolites were present in urine, which indicates that these molecules are not absorbed [47]. The beneficial health effects of EGCG could be attributed to its anti-oxidant activity and free radical chelation during the break down of ingested food. Furthermore, the stomach’s epithelial surface is known to be threatened by bacteria that can swim through the protective mucus layer, such as helicobacter pylori which causes ulcers [24]. By ingesting large amounts of EGCG containing beverages or food the modification of the mucus layer’s viscoelasticity could potentially provide a further defensive measure against these pathogens, since it will compromise the motility of the bacteria. The same conclusions can be extrapolated to the small intestinal mucus layer, which also hosts bacteria and acts as a selective barrier for nutrients. Modifications in the viscoelasticity and binding of broken down components of ingested food could contribute to the array of health benefits attributed to polyphenols.

## Conclusions

The interaction of polyphenols with gastric and duodenal mucin solutions was investigated using particle tracking microrheology and small-angle neutron scattering. Isolated EC and EGCG compounds were used to treat both Muc5ac and Muc2 mucin solutions to test the assumption that catechins which have a galloyl ring in their structure strongly interact with mucins, whereas catechins without a galloyl ring in their structure do not. Gelation of the mucin solutions occurred when concentrations of EGCG as low as 0.3% w/w were added. A very small change of the rheological characteristics of the solutions occurred when EC was added. This implies that the presence of the galloyl ring in the EGCG molecule allows it to bind to mucin, which leads to association and eventually to aggregation. These findings were also observed using SANS. Polyphenol induced mucin gelation was observed by a vastly increased intensity of the scattering profiles in the low-q range and the *n>3* fractal dimension that indicates a nanoscale phase separated gelled structure.

Additionally, using the same methods, green and black tea extracts were combined with mucin solutions. The weaker effect of black tea extract on the viscoelasticity of the mucin solutions further strengthens the aforementioned conclusion regarding EGCG, since its total polyphenol content was lower compared to the green tea extracts. Furthermore, the stronger effects of the EGCG-rich green tea extract compared to the crude green tea extract, allowed us to conclude that EGCG has the highest affinity to interact with mucins, causing precipitation and gelation, whereas EC and non-galloylated epicatechins do not.

## Supporting Information

File S1 (DOCX)
