# Supplementary material for: Tea Derived Galloylated Polyphenols Cross-Link Purified Gastrointestinal Mucins
Source: PLoS One. 2014 Aug 27;9(8):e105302. doi: 10.1371/journal.pone.0105302 (PMC4146515; doi:10.1371/journal.pone.0105302)
Supplement: File S1 — (DOCX) [file pone.0105302.s001.docx]

**Supporting Information**

**SI Text**

**Materials and methods:**

The list of the polyphenols used:

*Epigallocatechin gallate* (EGCG): Commercially available Teavigo, DSM Nutritional Products Inc., purified from green tea. The sample was found to be 94% w/w EGCG by HPLC.

*Epicatechin*: Isolated from green tea and it was found to be 98.4% w/w pure by HPLC.^1^

*Green tea extract*: The polyphenol content is 34.6% w/w and catechins 23.5% w/w. The main catechin was found to be EGCG (8.5% w/w).

*Black tea extract*: Total polyphenol content was found to be 30.2% w/w and EGCG was the most abundant catechin identified. Furthermore, 7.5% w/w was caffeine and 0.9% w/w gallic acid.

*EGCG rich green tea extract*: Commercially available Sunphenon 80SK green tea extract. Total polyphenol content was 77.02% w/w of which the total EGCG content was 38.28% w/w (quantified using HPLC).

**2.3 Small Angle Neutron Scattering (SANS)**

SANS2D is a time-of-flight small-angle scattering instrument, which uses thermal neutrons of wavelength in the range 1.75<λ<16.5 Å.

Due to the large size of mucin oligomers and the large aggregates formed upon treatment with polyphenols, the accessible q-range did not span into the Guinier regime, which would occur at lower q.

Appropriate normalisation using site-specific procedures allowed for the absolute cross section (I(q)) (cm^-1^) as a function of momentum transfer q (Å^-1^) to be calculated. Quartz Hellma cells (2 mm path length) were used to load the samples in the apparatus and the incident beam was set to have a diameter of 8 mm. Additionally, the scattering profile of the solvent and of the empty cell were measured and subsequently subtracted from the raw SANS data, so that the normalised scattering profile of the solution could be obtained. ^2–4^

**3. Results**

**3.3 Small Angle Neutron Scattering**

A fractal dimension of 1.72±0.01 was observed throughout the accessible q-range of the SANS scattering profiles of the untreated Muc5ac and Muc2 solutions, Fig 5.

**References**

(1) Rossetti, D.; Bongaerts, J. H. H.; Wantling, E.; Stokes, J. R.; Williamson, A.-M. *Food Hydrocoll.* **2009**, *23*, 1984–1992.

(2) Mantid project http://mantidproject.org.

(3) Wignall, G. D.; Bates, F. S. *J. Appl. Cryst.* **1987**, *20*, 28–40.

(4) Hammouda, B. *Polym. Rev.* **2010**, *50*, 14–39.

(5) Thornton, D. J.; Rousseau, K.; McGuckin, M. A. *Annu. Rev. Physiol.* **2008**, *70*, 459–486.

**Supporting Figures**


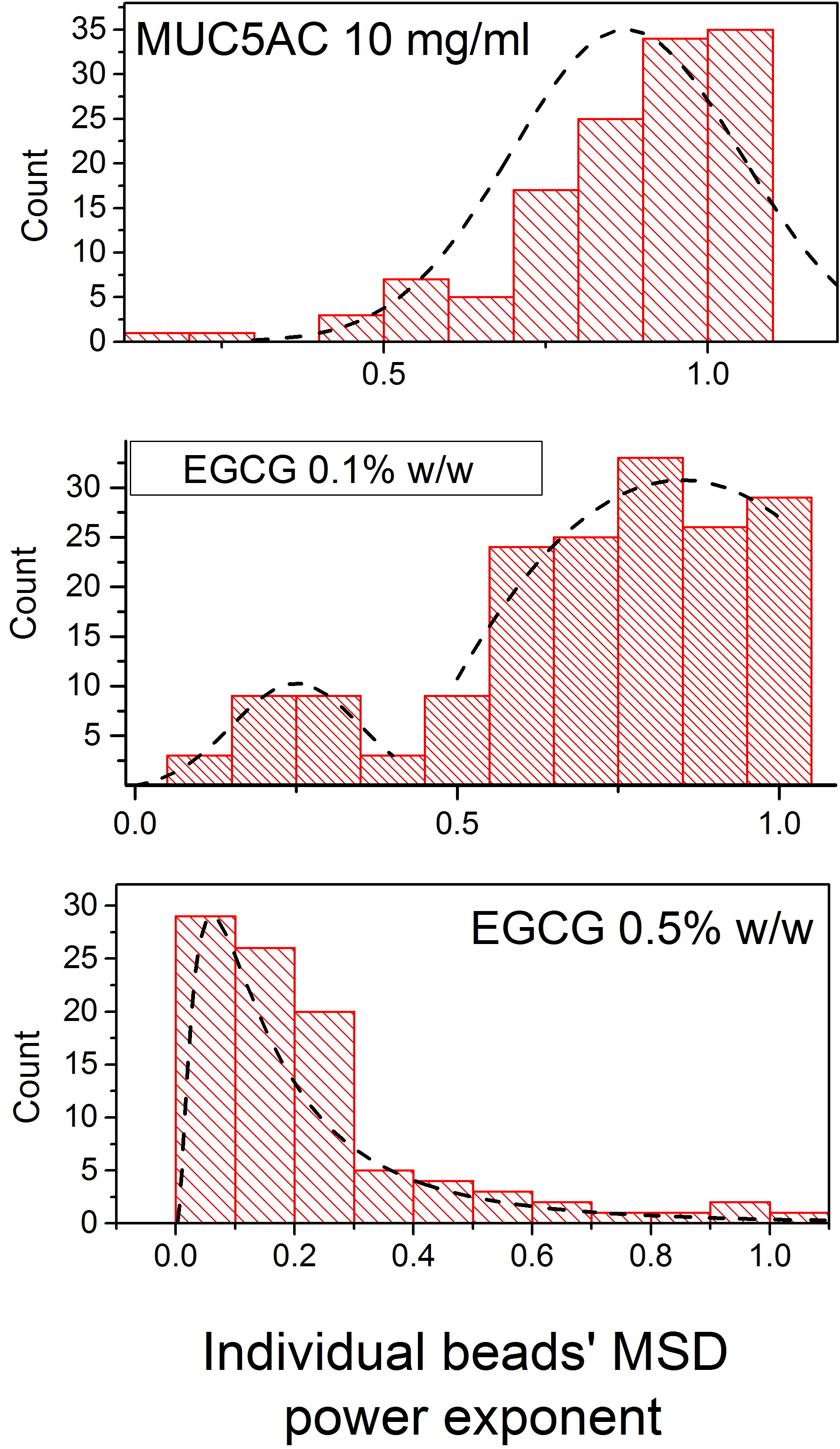


**Figure S1:** Histograms of individual beads’ MSD power exponents obtained from an untreated 10 mg/ml Muc5ac solution and 10 mg/ml Muc5ac solutions treated with 0.5% and 1% w/w EGCG. Dotted lines represented a fitted Normal distribution curve. The beads embedded in the purely viscous untreated solution are observed to be diffusing, shown by the peak of the curve towards 1. At 0.1% w/w, there are two populations of beads observed, one is diffusing and the other one is trapped in the gel network, shown by the two peaks at each end. Finally, at 0.5% w/w EGCG the solution has undergone a sol-gel transition, shown by the peak of the curve towards 0.


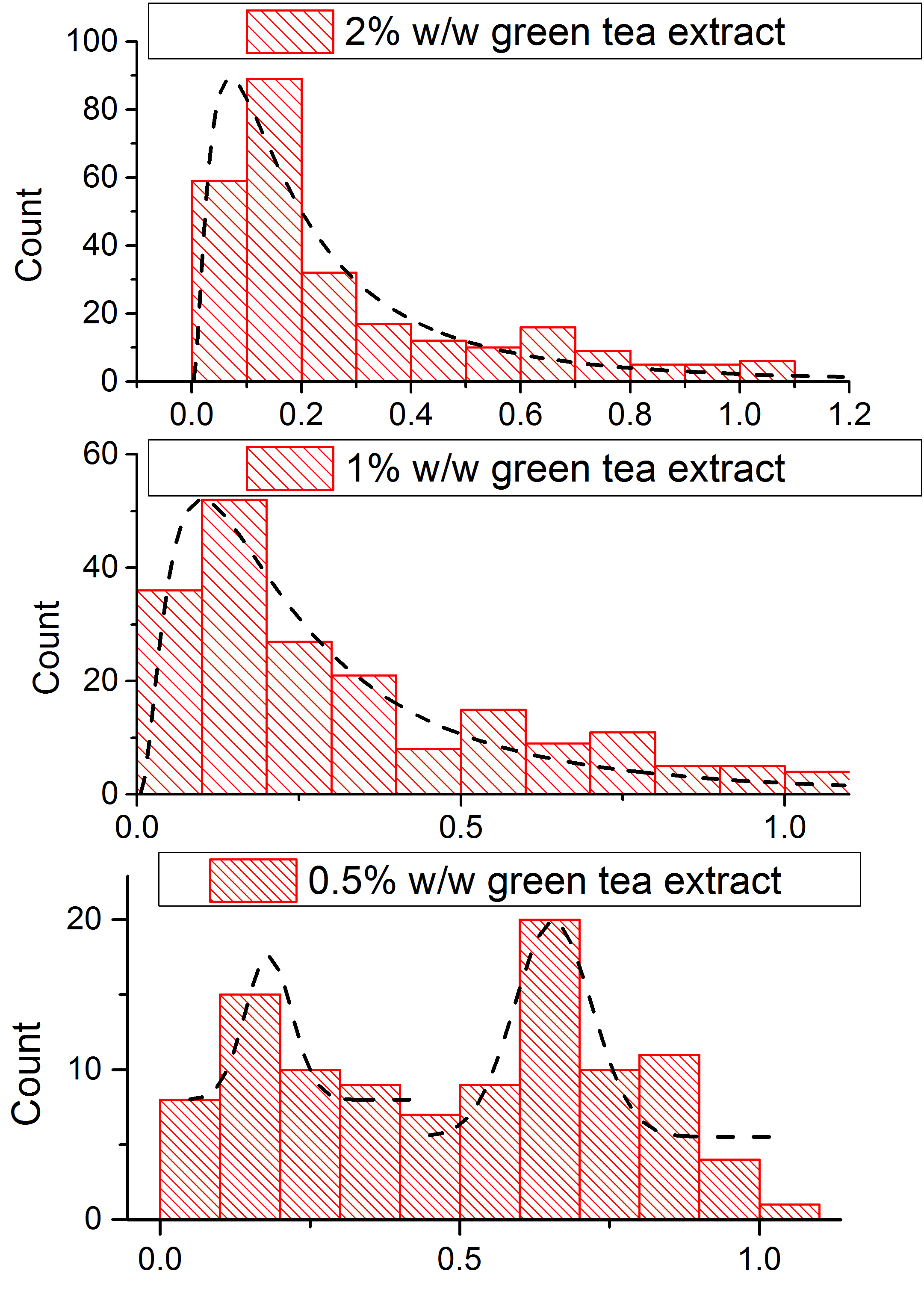


**Figure S2:** Individual bead’s MSD power exponents obtained from Muc5ac solutions treated with green tea extract. At 0.5% w/w of the mixture, there is a broad distribution of exponents, indicating the highly polydisperse nature of the formed network. There are two populations of beads observed, shown by the two peaks in the data; one is trapped in the gel network and the other diffusing in regions of lower viscosity. At 1% and 2% of the mixture, the distribution is skewed towards 0, indicating more beads are trapped in the gel network than those diffusing, as a result of the sol-gel transition.


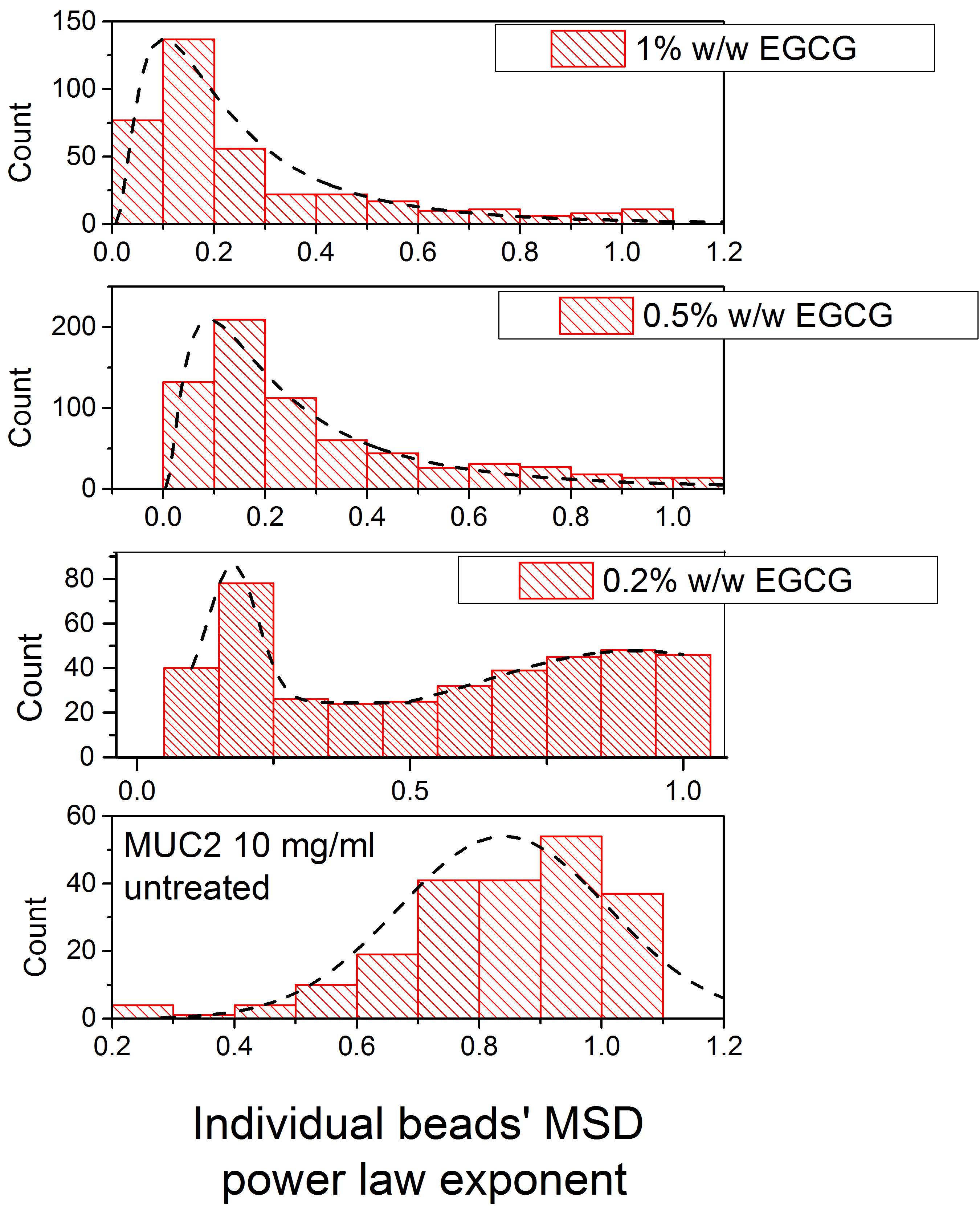


**Figure S3:** Individual beads’ MSD power exponents obtained from Muc2 solutions treated with EGCG. As with Muc5ac, the untreated 10 mg/ml Muc2 solution’s exponent distribution is skewed towards 1. At 0.2% w/w EGCG there is a large degree of heterogeneity observed, shown by the two distinct populations; one trapped in the gelled network (peak towards 0) and one diffusing in regions of lower viscosity (peak towards 1). At higher concentrations of EGCG the distribution is skewed towards 0 as a result of the majority of the embedded microspheres being trapped in the gel network.


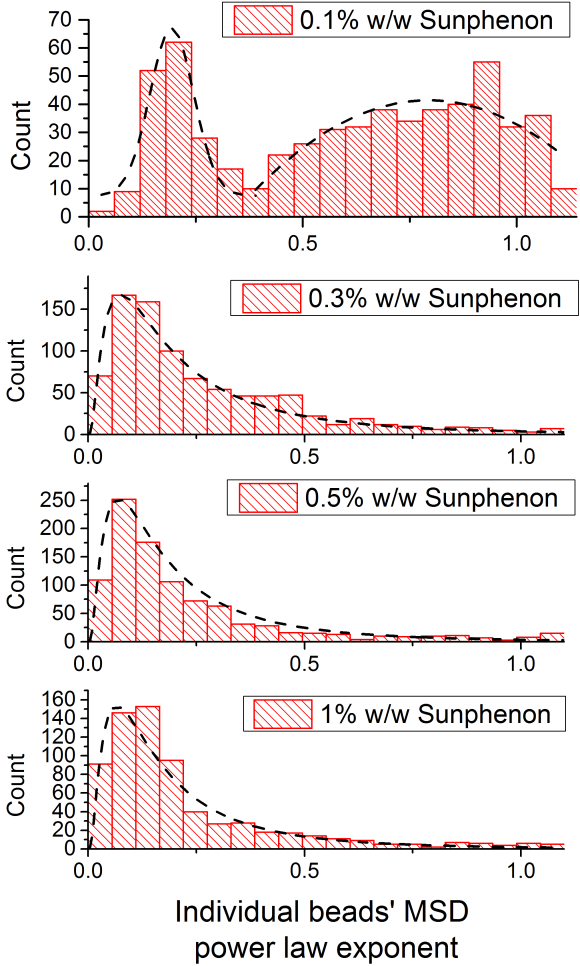


**Figure S4:** Histograms of individual beads’ MSD power exponents obtained from Muc2 solutions treated with Sunphenon 80SK green tea extract. At 0.1% w/w of the mixture, there are two distinct populations in the solution; one is diffusing in regions of lower viscosity (peak towards 1) and the other is trapped in the gel network (peak towards 0). As observed with PTM, the solution undergoes a sol-gel transition at 0.5% w/w Sunphenon, shown by the single peak in the distributions towards 0.


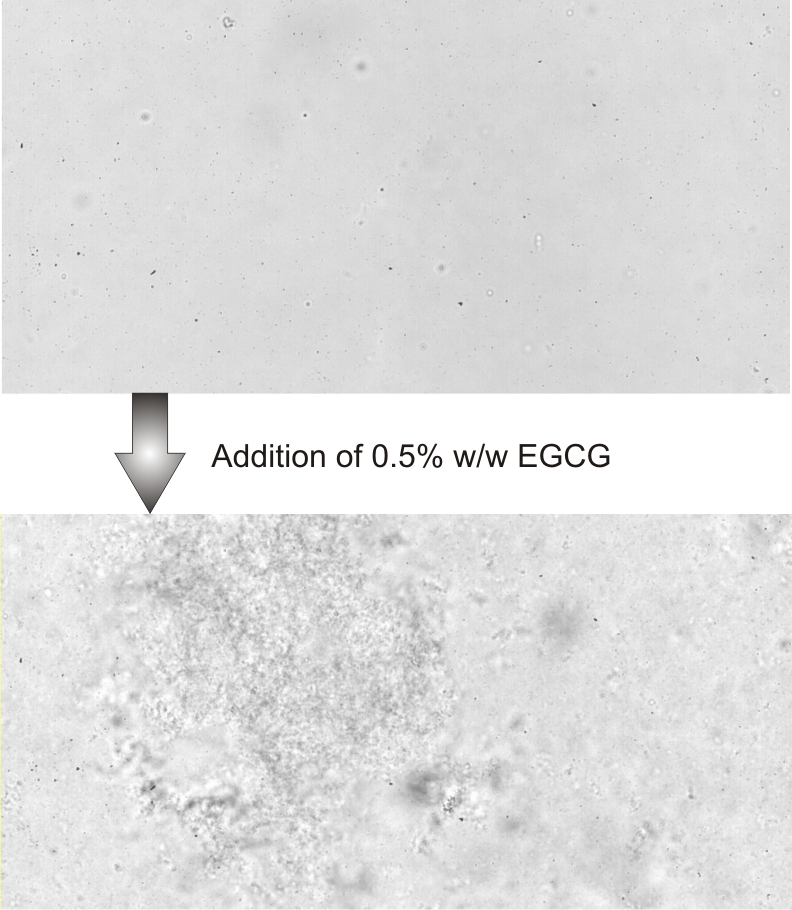


**Figure S5:** Bright field optical microscopy images of a 10 mg/ml Muc2 solution before and after the addition of 0.5% w/w EGCG; a sufficiently high concentration to induce a sol-gel transition. The complexation and aggregation of protein is immediately apparent after treatment, with large aggregates being observed throughout the sample. The embedded probe spheres are seen in both images as black dots.


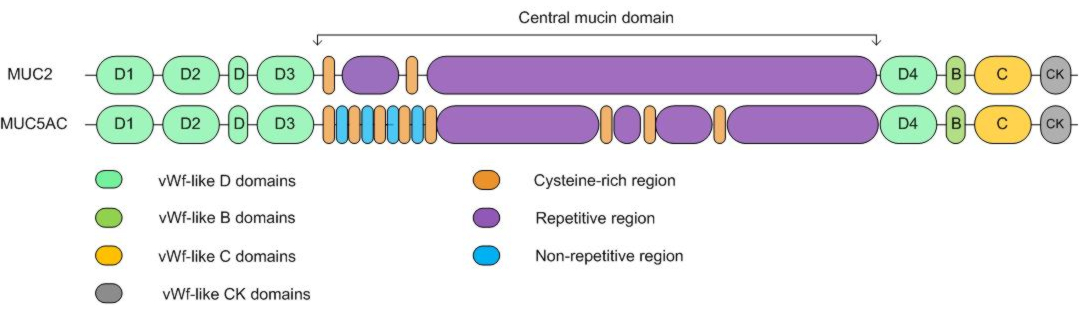


**Figure S6:** A schematic representation of the structure of Muc2 and Muc5ac. Both types of mucins share common N- and C- termini structure, with their main difference found along the mucin domain, where Muc2 has 2 cysteine-rich domains, whereas Muc5ac can have multiple (at least 8). This could provide more sites for EGCG binding and thus more sites for aggregation. [Image adapted from ^5^]


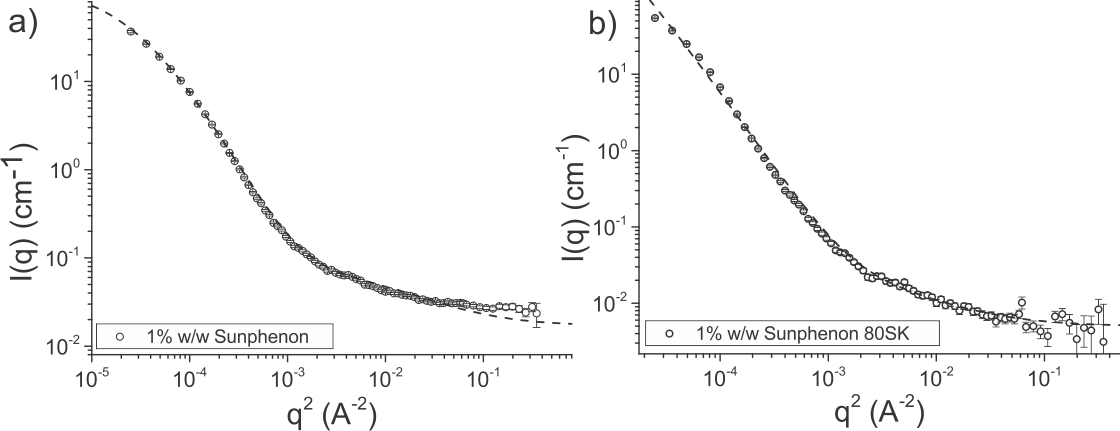


**Figure S7:** The scattering profiles obtained from 1% w/w Sunphenon 80SK treated (a) Muc5ac and (b) Muc2 solutions. The two-phase separated systems are well characterised by the combination of Ornstein-Zernicke and Debye-Bueche functions (equation 1 main text), suggesting the formation of large scale structures within the solution, i.e. the nanoscale phase separated gelled polymer network, in which the phase separation effects dominate the scattering at low-q.

| Sample | **Viscosity (mPas)** | **Elastic shear modulus (Pa)** | **Relaxation time (ms)** |
| --- | --- | --- | --- |
|  |  |  |  |
| 10 mg/ml Muc5ac | 12.0±0.3 | Negligible | - |
| 10 mg/ml Muc5ac + 0.5% w/w EC | 14.4±0.6 | Negligible | - |
| 10 mg/ml Muc5ac + 0.1% w/w EGCG | 58.2±1.4 | 0.37±0.03 | 157±9 |
| 10 mg/ml Muc5ac + 0.2% w/w EGCG | 431±6 | 1.01±0.12 | 427±51 |
| 10 mg/ml Muc5ac + 0.5% w/w EGCG | 1,670±120 | 1.58±0.12 | 1.06±0.09 |
| 10 mg/ml Muc5ac + 1% w/w EGCG | 2,120±160 | 1.48±0.08 | 1.43±0.13 |
|  |  |  |  |
| 10 mg/ml Muc5ac + 0.5% w/w green tea extract | 78.9±1.4 | 0.57±0.02 | 138±5 |
| 10 mg/ml Muc5ac + 1% w/w green tea extract | 214±2 | 0.95±0.02 | 225±5 |
| 10 mg/ml Muc5ac + 2% w/w green tea extract | 2,030±120 | 1.24±0.04 | 1.6±0.1 |
|  |  |  |  |
| 10 mg/ml Muc5ac + 0.5% w/w black tea extract | 42.2±0.6 | 0.69±0.01 | 61±1 |
| 10 mg/ml Muc5ac + 1% w/w black tea extract | 444±7 | 0.85±0.02 | 522±15 |
|  |  |  |  |
| 10 mg/ml Muc5ac + 0.1% w/w Sunphenon 80SK | 36.5±0.2 | 0.69±0.02 | 53±2 |
| 10 mg/ml Muc5ac + 0.3% w/w Sunphenon 80SK | 494±8 | 0.92±0.01 | 537±11 |
| 10 mg/ml Muc5ac + 0.5% w/w Sunphenon 80SK | 2,020±120 | 1.27±0.01 | 1,590±80 |
| 10 mg/ml Muc5ac + 1% w/w Sunphenon 80SK | 3,500±300 | 1.3±0.1 | 2,600±300 |

**Supplementary Table 1:** The viscoelastic properties of 10 mg/ml Muc5ac solutions treated with various types of polyphenols and tea extracts calculated with particle tracking microrheology. A direct correlation between the concentration of gallated polyphenols, such as EGCG, and the increase in the viscoelastic characteristics of the solutions is observed with all the compounds used. Above a threshold concentration, EGCG causes the Muc5ac solution to undergo a sol-gel transition, demonstrated by the large increase of the relaxation time of the sample to hundreds of ms, typical of weak physical hydrogels.

| Sample | **Viscosity (mPas)** | **Elastic shear modulus (Pa)** | **Relaxation time (ms)** |
| --- | --- | --- | --- |
|  |  |  |  |
| 10 mg/ml Muc2 | 6.7±0.3 | Negligible | - |
| 10 mg/ml Muc2 + 0.5% w/w EC | 8.9±0.4 | Negligible | - |
| 10 mg/ml Muc2 + 0.2% w/w EGCG | 271±3.4 | 0.80±0.02 | 340±10 |
| 10 mg/ml Muc2 + 0.5% w/w EGCG | 401±6 | 1.09±0.04 | 367±15 |
| 10 mg/ml Muc2 + 1% w/w EGCG | 923±19 | 1.16±0.03 | 796±26 |
| 10 mg/ml Muc2 + 0.5% w/w green tea extract | 65.2±4.2 | 0.77±0.08 | 84.6±10.3 |
| 10 mg/ml Muc2 + 1% w/w green tea extract | 155±2 | 0.88±0.02 | 176±4 |
| 10 mg/ml Muc2 + 2% w/w green tea extract | 287±5 | 1.22±0.02 | 236±6 |
| 10 mg/ml Muc2 + 0.5% w/w black tea extract | 64.3±0.4 | 0.58±0.01 | 111±2 |
| 10 mg/ml Muc2 + 1% w/w black tea extract | 146±1 | 0.66±0.02 | 221±7 |
| 10 mg/ml Muc2 + 0.1% w/w Sunphenon 80SK | 13.1±0.1 | 0.71±0.03 | 18±1 |
| 10 mg/ml Muc2 + 0.5% w/w Sunphenon 80SK | 387±7 | 0.70±0.02 | 553±19 |
| 10 mg/ml Muc2 + 1% w/w Sunphenon 80SK | 489±8 | 0.95±0.01 | 515±10 |
| 10 mg/ml Muc2 + 2% w/w Sunphenon 80SK | 1,300±80 | 1.12±0.08 | 1,160±120 |

**Supplementary Table 2:** The viscoelastic properties of 10 mg/ml Muc2 solutions treated with various types of polyphenols. As with Muc5ac, there is a direct correlation between the concentration of gallated polyphenols and the viscoelasticity of the sample, with Muc2 solution undergoing a sol-gel transition above a certain threshold.

| Sample | **$I_{1}(0)$**(cm^-1^) | ξ (Å) | **$I_{ex}(0)$**(cm^-1^) | Ξ (Å) |
| --- | --- | --- | --- | --- |
| Muc5ac + 1% w/w Sunphenon | 0.05±0.01 | 8.5±0.4 | 124±5 | 176±3 |
| Muc5ac + 1% w/w EGCG | 0.03±0.01 | 14.1±1.8 | 128±9 | 164±4 |
| Muc5ac + 1% w/w black tea extract | 0.04±0.01 | 12±1 | 24±3 | 97±3 |
| Muc5ac + 1% w/w green tea extract | 0.08±0.01 | 22±2 | 62±4 | 121±4 |
|  |  |  |  |  |
| Muc2 + 0.5% w/w EGCG | 0.010±0.002 | 10±1 | 4104±250 | 522±129 |
| Muc2 + 1% w/w EGCG | 0.010±0.002 | 6.8±4 | 3075±850 | 466±90 |
| Muc2 + 1% w/w Sunphenon | 0.010±0.002 | 9±2 | 6150±2100 | 558±188 |
| Muc2 + 1% w/w black tea extract | 0.08±0.01 | 6±2 | 80±3 | 153±2 |
| Muc2 + 1% w/w green tea extract | 0.09±0.02 | 20±6 | 310±40 | 225±9 |

**Supplementary Table 3:** The parameters calculated from SANS experiments which characterise the two-phase separated mucin solutions after treatment with phenolic compounds. Muc2 solutions were observed to undergo a complete phase transition after addition of polyphenols, demonstrated by the higher I_ex_(0) and the Porod exponents of the low-q scattering intensity graph. The parameters were calculated from a fit of equation (5)
